# Hepatic, Extrahepatic and Extracellular Vesicle Cytochrome P450 2E1 in Alcohol and Acetaminophen-Mediated Adverse Interactions and Potential Treatment Options

**DOI:** 10.3390/cells11172620

**Published:** 2022-08-23

**Authors:** Santosh Kumar, Bhupesh Singla, Ajay K. Singh, Stacey M. Thomas-Gooch, Kaining Zhi, Udai P. Singh

**Affiliations:** 1 Department of Pharmaceutical Sciences, College of Pharmacy, The University of Tennessee Health Science Center, Memphis, TN 38163, USA; 2 Plough Center for Sterile Drug Delivery Solutions, The University of Tennessee Health Science Center, Memphis, TN 38163, USA

**Keywords:** alcohol, acetaminophen, extracellular vesicles, nutraceutical, drug interaction, hepatic cells, toxicity

## Abstract

Alcohol and several therapeutic drugs, including acetaminophen, are metabolized by cytochrome P450 2E1 (CYP2E1) into toxic compounds. At low levels, these compounds are not detrimental, but higher sustained levels of these compounds can lead to life-long problems such as cytotoxicity, organ damage, and cancer. Furthermore, CYP2E1 can facilitate or enhance the effects of alcohol-drug and drug-drug interactions. In this review, we discuss the role of CYP2E1 in the metabolism of alcohol and drugs (with emphasis on acetaminophen), mediating injury/toxicities, and drug-drug/alcohol-drug interactions. Next, we discuss various compounds and various nutraceuticals that can reduce or prevent alcohol/drug-induced toxicity. Additionally, we highlight experimental outcomes of alcohol/drug-induced toxicity and potential treatment strategies. Finally, we cover the role and implications of extracellular vesicles (EVs) containing CYP2E1 in hepatic and extrahepatic cells and provide perspectives on the clinical relevance of EVs containing CYP2E1 in intracellular and intercellular communications leading to drug-drug and alcohol-drug interactions. Furthermore, we provide our perspectives on CYP2E1 as a druggable target using nutraceuticals and the use of EVs for targeted drug delivery in extrahepatic and hepatic cells, especially to treat cellular toxicity.

## 1. Introduction

A recent report from the Centers for Disease Control and Prevention (CDC) showed approximately 100,306 drug overdose-related deaths in one year (2020–2021), which indicates an increase of 28.5% from the previous year [1]. Many drug overdoses, such as acetaminophen (APAP) cases, are accidental and due to inappropriate use, lack of education or false perception of safety, or easy accessibility of the product [2]. Millions of people self-medicate with APAP routinely and are uninformed that many combination products also contain APAP, which increases the risk of an overdose. Other APAP-associated risks (e.g., hepatotoxicity) are further exacerbated with the concomitant use of alcohol, especially in those who have existing liver damage [3]. Liver damage is caused by chronic abuse (≥6 months), heavy drinking (>14 drinks for men and >7 drinks for women per week), binge drinking (>6 drinks in one sitting), or APAP overdose (>4 g/day) [3,4]. Both APAP and alcohol are metabolized by cytochrome P450 2E1 (CYP2E1), which plays an important role in inducing hepatotoxicity caused by both alcohol and APAP [5].

CYP2E1, one of the major metabolizing enzymes in humans, is mainly expressed in the liver [6]. To a lesser extent, CYP2E1 has been found in extrahepatic systems such as lung microsomes (10 pmol of P450 per mg protein), neuronal cells, and kidney microsomes (expression varies with age) [7,8,9]. In other studies, CYP2E1 mRNA and/or protein expression has shown to be induced two to three-fold in lymphocytes, monocytes, macrophages, microglia and astrocytes, with alcohol exposure [10,11,12,13,14]. The involvement of CYP2E1 in the metabolism of alcohol, xenobiotic drugs, such as APAP, and organic compounds (halothane, enflurane, and isoflurane) can increase the risk of carcinogenic or toxic effects with repeated, improper, or abused use [15,16]. 

Although alcohol is primarily metabolized by alcohol dehydrogenase (ADH) to toxic acetaldehyde, it is the activation of the microsomal ethanol oxidizing system (MEOS), a secondary alcohol-metabolic pathway, that further causes cytotoxicity, organ damage, and cancer when CYP2E1 converts alcohol into acetaldehyde when ADH is saturated [17,18,19]. Additionally, like ADH-mediated alcohol metabolism, CYP2E1-mediated alcohol metabolism produces reactive oxygen species (ROS) and acetaldehyde in the liver, which subsequently cause DNA oxidation, lipid peroxidation, and protein oxidation, ultimately leading to liver damage, cirrhosis, and liver cancer [19,20].

In the era of the COVID-19 pandemic, there have been increasing reports of alcohol consumption and self-medication [21,22]. Since APAP is a substrate of CYP2E1, drug-drug and alcohol-drug interactions are common among individuals who routinely use APAP and/or are classified as chronic, heavy, or binge drinkers. Chronic use of APAP at a high dose (>4 g/day) is known to cause liver toxicity, and even death, in children due to the acute accumulation of its toxic metabolite, N-acetyl-p-benzoquinone imine (NAPQI) [3,4,23]. Although NAPQI is cleared quickly by glutathione conjugation, its acute accumulation even at 5% is toxic and causes damage. Furthermore, concurrent use of APAP and alcohol increases the risk of hepatic- and extrahepatic cytotoxicity [24]. Additionally, various disease conditions (diabetes, fasting, and obesity) alter the mRNA expression of CYP2E1 [25,26], which could potentially increase the chances of drug-drug and drug-alcohol interactions. However, subsequent studies have shown an increase in mRNA and protein expression of CYP2E1, but not its activity, in obese and/or diabetic animal models [26,27,28]. Interestingly, these studies showed decreased expression of most CYP enzymes. These findings suggest that further studies are needed to evaluate the role of CYP2E1 in drug-drug or drug-alcohol interactions in an obese/diabetic condition. Currently, N-acetylcysteine is the only therapeutic antidote for APAP overdose with added benefits (i.e., improved hemodynamics, oxygen use, and low side effect profile). Although the relative expression of CYP2E1 in extrahepatic organs is much lower than liver, various studies have shown the effects of xenobiotics, especially alcohol, on CYP2E1-induced toxicity in extrahepatic cells [12,13,14,29]. However, being a microsomal/membrane-bound enzyme, CYP2E1 cannot circulate freely in plasma. Interestingly, the discovery of extracellular vesicles (EVs) secreted from various cell types, which are found in biofluids (i.e., plasma, urine, milk, and saliva) that carry biomolecules [30,31,32], suggests that EVs may have a role in packaging and circulating CYP2E1 in plasma. To further corroborate this idea, Kumar and other groups have recently shown substantial expression of functional CYPs, including CYP2E1, in human plasma [33,34,35,36]. However, it is yet to be determined whether packaging of CYP2E1 in EVs and its secretion in plasma has a physiological role, or it is released into plasma as a result of hepatocyte death. Additionally, the ubiquitous expression of CYP2E1 makes it an important target for APAP metabolism and drug interactions, not only in the liver but also in extrahepatic locations, especially in the brain since antidotal therapy for alcohol- and drug-drug interactions do not readily cross the blood-brain barrier (BBB). 

In this review article we first discuss the roles of CYP2E1 in the metabolism of alcohol and drugs, facilitating cellular injury/toxicities, and interactions involving drugs/alcohol metabolism. Next, we review nutraceuticals that have shown potential in decreasing or averting alcohol-/drug-induced toxicity, then we highlight provisional outcomes of alcohol/drug-induced toxicity and potential treatment strategies. Finally, we cover the role and implications of EVs containing CYP2E1 in hepatic and extrahepatic cells during drug/alcohol interactions. 

## 2. The Role of Hepatic CYP2E1 in Causal Toxicity and Potential Treatment Options

In this section, we review in vitro and in vivo animal studies that have investigated the causal effects of hepatic CYP2E1 mRNA, protein, and/or activity on alcohol- and drug-induced hepatoxicities and liver damage. The expression of CYP2E1 mRNA, protein, and/or enzyme activity are not always correlated with each other. Subsequently, we discuss various nutraceutical compounds tested to prevent or treat hepatotoxicity and liver damage. The studies with nutraceuticals are important because disulfiram, and DAS, 4-methylpyrazole are non-specific inhibitors of CYP2E1 inhibitors, and have other targets/effects. 

### 2.1. Acetaminophen (APAP)-Induced Liver Toxicity/Injury

APAP is a commonly used analgesic found in mono and combination formulations and thus is easily overdosed, especially with improper use or duplicate therapy. This explains why it is a common cause of acute liver injury [37,38,39]. Further, the COVID-19 pandemic has amplified reports of APAP drug-induced liver injury, as it is used for managing symptoms [40]. Although the majority of APAP is quickly detoxified, a small amount of APAP metabolite, NAPQI, binds to GSH or proteins. While binding of NAPQI to GSH leads to phase II metabolic clearance, its binding to protein causes loss of protein functions leading to necrosis or apoptosis [41,42]. APAP-induced liver injury significantly increases ROS, hepatic malondialdehyde (MDA), serum aspartate transaminase, alanine transaminase, and lactate dehydrogenase levels [43,44]. Approximately 90% of APAP is excreted by sulfation and glucuronidation pathways, and 5–10% is removed in the form of NAPQI [37,45]. In obese COVID-19 patients with metabolic dysfunction-associated fatty liver disease (MAFLD), Ferron et. al., reported increased activity of CYP2E1, thus creating another complexity in the treatment regimen of these patients leading to an increased risk of drug-induced liver injury [46].

A study conducted by Licong et al. found that fisetin (FST), a flavonoid, reverses APAP-induced toxicity and reduces ROS in human fetal hepatocytes (L-02 cells) and male C57 mice [47]. The Jiang group studied APAP-induced liver injury in murine models that were treated intragastrically with Artemisia (Eucalyptol), an essential oil. Eucalyptol, an inhibitor of Kelch-like ECH-associated protein 1 (Keap1), activates nuclear factor erythroid 2-related factor 2 (Nrf2) and inhibits CYP2E1 activity leading to the suppression of NAPQI formation [38,43]. Another natural compound, shikonin (nutraceutical from the roots of Lithospermum erythrorhizon plants) in the study by Guo et al., was injected intraperitoneally to investigate its effects on APAP-induced toxicity in alpha mouse liver 12 cells and Balb/c mice [44]. Moreover, the anti-inflammatory and antioxidant properties of shikonin decreased interleukin (IL-6 and IL-1β), tumor necrosis factor-α (TNF-α), and CYP2E1 mRNA expression [44]. When APAP-challenged human hepatocellular carcinoma cells (HepG2) were treated with red betel leaf extract (from Indonesia) at 25 and 100 μg/mL, Ginting et al., showed hepatoprotective effects with elevated CYP2E1 and glutathione peroxidase gene expression [48]. Choi et al. investigated the effects of the natural alkaloid, rutaecarpine, on APAP-induced liver injury in mice [49]. Toxicity in mice was induced via intraperitoneal injection of APAP followed by oral administration of rutaecarpine; it decreased CYP2E1 protein expression and inhibited the expression of inflammatory cytokines by reducing nuclear factor kappa B cell (NF-κB) expression. Furthermore, diallyl disulfide (DADS) was used to suppress APAP-induced chronic liver injury by inhibiting CYP2E1 protein expression, anti-inflammatory, and antiapoptotic effects by inhibition of NF-κB, thus showing it to be protective [50]. 

### 2.2. Alcohol-Induced Liver Toxicity/Injury

The metabolism of alcohol by hepatic CYP2E1 leads to ROS production, consequently generating damaging toxic effects in the liver through mitochondrial dysfunction, DNA damage, and lipid peroxidation in a chronic-binge mice model [51]. Enhanced superoxide and H_2_O_2_ production are mainly due to poor coupling of CYP2E1 with NADPH-cytochrome P450 reductase (CPR) [52]. ROS influence posttranscriptional modifications through small ubiquitin-like modifications that covalently attach to epsilon-amino groups of lysine residues and modulate protein stability, activity, and localization [53]. To prevent inflammatory and oxidant responses seen in alcohol-induced liver injury, Han et al. administered astaxanthin, a xanthophyll carotenoid compound, to mice, which blocked STAT3, reduced ROS and decreased CYP2E1 protein expression [51]. HepG2 cells were pretreated with gastrodin for 4 h prior to ethanol treatment in a study by Zhang et al. The authors reported nullification of ethanol-induced toxicity and apoptosis with gastrodin treatment. Furthermore, in mice with ethanol-induced acute liver injury, gastrodin decreased the enhanced alcohol-mediated induction of both protein and enzyme activity of ADH and CYP2E1 [54]. In an intragastric ethanol-fed mouse model, ubiquitin-conjugation enzyme 9 was expressed in the liver; silencing this enzyme by siRNA lowered CYP2E1 mRNA and protein expression and prevented ROS production involved in alcoholic liver disease (ALD) [53]. In the Nagappan et al., study with chronic ethanol-fed mice, the use of cryptotanshinone from Salivamiltiorrhiza Bunge plants protected hepatocytes from ethanol-induced acute liver injury by inhibiting oxidative stress and lipogenesis [55]. Cryptotanshinone conferred its hepatoprotective effects through the activation of the AMP-activated protein kinase (AMPK), sirtuin 1 (SIRT1) as well as Nrf2 and the inhibition of CYP2E1 pathways. Recently, Avila and coworkers discussed the benefits of inhibiting overexpressed CYP2E1 protein levels for the treatment of ALD [56]. In mouse models, pharmacological inhibition of CYP2E1 with clomethiazole and genetic deletion of CYP2E1 partly prevented ALD [57,58]. In contrast, CYP2E1 overexpression enhanced the severity of ALD, suggesting the detrimental role of CYP2E1 activity in alcohol-induced liver damage [59].

The N-terminal signal of CYP2E1 protein regulates its targeting to mitochondria [60]. CYP2E1 N-terminal signal variant W23R/W30R preferentially targets CYP2E1 to mitochondria and poorly targets it to the endoplasmic reticulum [61]. Mitochondrial CYP2E1 stimulates significantly higher alcohol-induced ROS generation and induces cell injury in comparison to microsomal CYP2E1 [61]. Further, previous investigations have shown that mitochondrial CYP2E1 promotes direct damage to mitochondrial cytochrome c oxidase, which can be rescued with mitochondrial antioxidant treatment [61,62], suggesting the role of mitochondrial CYP2E1 in alcohol-induced mitochondrial ROS production and hepatotoxicity.

### 2.3. Drug-Drug Interactions

Multi-drug combinations are given to increase the therapeutic efficacy and success of drugs in treatment regimens of different diseases and comorbidities. However, these same drug combinations can also lead to unfavorable outcomes such as toxicity, treatment failure, or adverse drug interactions [63,64]. Drug-drug interactions (DDI) occur when a drug inhibits or induces the level of a cytochrome CYP enzyme, especially the major drug-metabolic CYP3A4, which causes an alteration in the metabolism of another drug [65,66,67]. Inhibition of CYP enzymes can cause decreased metabolism of other substrates leading to increased drug plasma concentration and eventually drug-induced toxicity [68,69]. Induction of CYP enzymes by a drug causes increased metabolism of other substrates leading to suboptimal plasma drug concentration or elevated levels of drug metabolites, which can lead to metabolite-induced toxicity and reduced drug efficacy [68].

A study conducted by Liu et al. on rats, using APAP and roxithromycin in combination, showed the induction of CYP2E1 mRNA and protein, enhanced levels of NAD(P)H, quinone oxidoreductase 1 (NQO1), TNFα, malondialdehyde, and lower mRNA and protein expression of glutathione peroxidase and superoxide dismutase (SOD) [63]. They also showed an increased mRNA and protein expression of CYP2E1 resulting in oxidative stress-mediated liver damage. In another study with 10-week-old C57BL/6J mice, the co-administration of valproic acid (VPA) and APAP showed an increase in severity of liver damage consistent with high expression of CYP2E1 mRNA, the presence of NAPQI-protein, and depletion of glutathione in the liver [70]. This study also revealed upregulated expression of steroidogenic acute regulatory protein 1 (STARD1) with endoplasmic reticulum (ER) stress-causing hepatotoxicity mediated by phosphorylation of c-Jun N-terminal kinase (JNK1/2) and SH3BP5 (SH3 domain-binding protein 5 also called SAB). Furthermore, administration of N-acetylcysteine (NAC; 2.5 mmol/kg IP) demonstrated protection against VPA- and APAP-induced liver toxicity by preventing ER stress and STARD1 induction. Cepharanthine, a biscoclaurine alkaloid, has been shown to prevent DDI via CYP2E1 in vitro human liver microsomes [71]. Anemarsaponin BII (ABII) is an active saponin and competitive inhibitor of CYP2E1 activity with half-maximal inhibitory concentration (IC50) and dissociation constant (Ki) values of 19.72 μM and 9.82 μM, respectively, suggesting a DDI between ABII and drugs metabolized by CYP2E1 [72]. A study conducted on rats and hepatic cells revealed that anti-tuberculosis drugs, such as rifampicin and isoniazid, induce liver injury by regulating the nucleotide-binding domain (NOD)-like receptor protein 3 (NLRP3) inflammasome and enhancing CYP2E1 mRNA expression [73].

### 2.4. Alcohol-Drug Interactions

Heavy alcohol consumption with other drugs leads to liver damage stemming from the generation of superoxide anion radicals and hydrogen peroxide after CYP2E1 metabolism. [74]. In alcohol-drug interactions, CYP2E1 is regulated by NF-κB, which activates other factors involved in liver injuries, such as inducible nitric oxide synthase, TNF-α, and IL-1β [75]. Chlormethiazole, a sedative drug, and certain phytochemicals (phenethyl isothiocyanate and sulforaphane), are used as potent inhibitors of CYP2E1 for protection and treatment of liver damage in alcohol consumers [76]. A study by Jiang et al. in mice showed that compounds such as isoquercetin (50 mg/kg), hyperoxide (50 mg/kg), 3-hydroxyphenyl acetic (50 mg/kg), 4-hydroxyphenyl acetic acid (50 mg/kg), and 3,4-hydroxyphenyl acetic acid (50 mg/kg) inhibit the protein expression of CYP2E1 and protect the liver from alcohol-induced (50%, 15 mL/kg) and APAP-induced (300 mg/kg) liver damage [77]. In chronic alcohol-drinking rodent models, simulating liver injury, the administration of CYP2E1 inhibitors chlormethiazole, phenethyl isothiocyanate, and diallyl sulfide (DAS) reduced lipid peroxidation and alcohol-induced oxidative stress [74,78]. 

## 3. Extrahepatic CYP2E1 in Drug Interactions and Potential Treatments

In this section, we discuss the role of CYP2E1 in alcohol-induced and drug-induced toxicity in extrahepatic cells, and also organ damage, as well as drug-drug and alcohol-drug interactions in vitro and in vivo animal studies. Further, this section comprises a discussion on the nutraceuticals used in various studies to prevent or treat extra-hepatotoxicity and organ damage.

### 3.1. Kidney

In addition to liver damage, drug overdose and/or adverse drug interactions, such as with APAP, also cause kidney injury, especially nephrotoxicity [79]. Nephrotoxicity induced by APAP is characterized by renal tubular cell apoptosis via up-regulation of CYP2E1, NF-κB, TNF-α, and cyclooxygenase-2 [50]. Akakpo et al. showed that 4-methyl pyrazole inhibits APAP-induced nephrotoxicity (300 to 600 mg/kg) in C57BL/6 mice by suppressing NAPQI [80]. Data from this study also showed that 4-methylpyrazole prevents APAP-induced nephrotoxicity by reducing oxidative stress in renal tubular epithelial cells and decreasing mitochondrial dysfunction. Another study showed that the mitochondrial complex I inhibitor, rotenone, protects the kidney against APAP-induced injury through the inhibition of mitochondrial oxidative stress and inflammation [81].

Cheng et al., injected lipopolysaccharide (LPS) intraperitoneally into mice to induce acute kidney injury (AKI). AKI mice were then treated with romidepsin (FK228) or depsipeptide; both compounds downregulated the expression of CYP2E1 by blocking the binding of hepatocyte nuclear factor-1 alpha (HNF-1α) with CYP2E1 promoter, thus reducing nephrotoxicity [82]. In addition, Un et al., delivered a single dose of 10 mg/kg (IP) cisplatin, a chemotherapeutic drug, to rats to elicit nephrotoxicity. The cisplatin-induced hepatotoxicity and nephrotoxicity revealed an elevation in creatinine, blood urea nitrogen, tissue cytokines (NF-κB and TNF-α), and oxidative stress (via CYP2E1). Aprepitant was subsequently given orally to rats in different doses (5, 10, and 20 mg/kg) and all increments conferred nephroprotective effects in addition to innate anti-emetic effects [83].

### 3.2. Monocytes, Astrocytes and Neuronal Cells

Brain CYP enzymes cause an immediate impact on neurotoxicity and drug response through different metabolic mechanisms in the brain [84,85,86]. Each region and cell type of the brain has a different metabolic capacity due to functional heterogeneity [87]. Overall, the CYP content in the brain is around 0.5%–2% of hepatic CYP content [86]. Even with this seemingly low amount in the brain, due to the low level of ADH, the role of CYP2E1 was shown to be greater than ADH in alcohol metabolism [14]. Yu et al., in ischemia/reperfusion injury mouse model, revealed the role of CYP2E1 in the BBB integrity as measured by 20-hydroxyecosatetraenoic acid (20-HETE) activity and BBB transfer rate [88]. In this study, CYP2E1 knock-out mice showed lower production of ROS, reduced apoptosis, neurodegeneration, lesions, and fewer neurological issues compared to control mice. These findings were consistent with the increased 20-HETE level, decline in BBB damage, increased inflammatory cytokines and increased microglia activation.

U937 monocytic cell lines and primary cells derived from peripheral blood mononuclear cells (PBMCs) are model cells to study many diseases including HIV-related pathogenesis, especially in the presence of drug abuse including alcohol. Alcohol consumption is highly prevalent in the HIV population compared to the general population [89,90]. Furthermore, Rao and Kumar showed an interaction between alcohol and anti-HIV drugs leading to increased oxidative stress and cytotoxicity [29]. Though 10-fold to 20-fold lower than hepatic CYP2E1, the Kumar group showed substantial expression of CYP2E1 in monocytic U937 cells and primary macrophages, which was further induced (two to three-fold) by alcohol exposure [12,13]. The group further showed that CYP2E1 is induced by alcohol via ROS-mediated induction of protein kinase C/c-Jun N-terminal kinase/specificity protein1 (PKC/JNK/SP1) pathway, which ultimately causes apoptotic cell death [14]. Furthermore, SVGA astrocyte cell death was prevented by using DAS, antioxidants (vitamin C and E), and CYP2E1 siRNA. In a separate study, treatment with 2-chloroethanol induced the expression of CYP2E1 in rat brain and caused cytotoxicity in rat astrocytes with an enhanced level of ROS, MDA, and apoptotic cells. Increased levels of CYP2E1, ROS, MDA, apoptotic cells, and phosphorylated ERK1/2 (extracellular signal-regulated kinase1/2) were attenuated in rat astrocytes when pretreated with either diallyl sulfide (DAS) or vitamin C prior to exposure of 2-chloroethanol [91].

### 3.3. Tumor Cells

Heterocyclic amines such as 2-amino-1-methyl-6-phenylimidazo [4,5] pyridine (PhIP) and alcohol-induced breast cancer, showed elevation in CYP2E1 expression and enhanced ROS generation through the estrogen-receptor-α (ER-α)-STAT-3 pathway in human breast adenocarcinoma cell lines (MCF-7; ER-α+) [92]. Further, CYP2E1-mediated oxidation of APAP drives higher proliferation and migration of breast cancer cells with high estrogen receptor expression [93,94]. Moreover, ICI 182780 (faslodex), 4’-hydroxytamoxifen, and antiestrogens were shown to prevent APA-induced proliferation in estrogen receptor +/progesterone receptor + cells [82]. Additionally, Xu et al. revealed that ganoderic acid, derived from Ganoderma mushrooms, may have the potential to prevent CYP2E1-induced cancers and other diseases in alcohol drinkers [95]. In this study the authors showed that ganoderic acid inhibits several CYP isoforms, including CYP2E1, in human liver microsomes, resulting in reduced alcohol-mediate oxidative stress and potential prevention of certain cancers.

## 4. CYP2E1 in Clinical Drug-Drug and Alcohol-Drug Interactions

### 4.1. Genotype of CYP2E1 and Related Drug Interactions

Genotype sequencing is a commonly used and helpful tool to predict potential drug interactions in clinical research and practice [96]. Popular sequencing methods include polymerase chain reaction (PCR) [97], TaqMan assays, and the SNaPshot analysis method [98]. Vuilleumier et al. used the genotype sequencing method to investigate the role of CYP2E1 in isoniazid-induced hepatoxicity in patients with dormant tuberculosis. They found a positive correlation in patients with the CYP2E1*1A/*1A genotype with the rate of developing isoniazid-induced hepatotoxicity without hepatitis [96]. Further, Costa et al. expanded the research of Vuilleumier et al. to target CYP2E1-induced adverse effects. These studies confirmed that AT-variants of CYP2E1 correlates with a higher capacity to metabolize drugs, which results in adverse effects [97]. It should be noted that post-transcriptional changes have been shown to regulate CYP2E1 protein expression in human liver. Liao et al. demonstrated an association between SNP 1561A > G present in CYP2E1 3′-UTR and reduced CYP2E1 mRNA levels in human PBMCs [99]. Various single nucleotide variants of CYP2E1 and their association with different pathological disorders have been recently reviewed [100]. Further, the CYP2E1 genotype has been shown to affect APAP and alcohol metabolism. Ueshima et al. examined APAP metabolism in subjects carrying different CYP2E1 genotypes and found that in healthy individuals, type A (c1/c1) and type C (c2/c2) correlate with longest and shortest APAP half-life in blood, respectively [101]. Further, in alcoholic noncirrhotic patients with type B, APAP half-life reduces within one week after abstinence compared to that in alcoholic patients with type A and normal subjects with type B. Additionally, a study has reported that 84% ALD patients harbor the c2 genotype, indicating an association between CYP2E1 polymorphisms and development of ALD [102].

### 4.2. Probe Substrates and Drug Interactions

The metabolic process of chlorzoxazone through CYP2E1 and the formation of 6-hydroxychlorzoxazone, (detectable in human plasma or urine) is used to assess the enzymatic activity of CYP2E1 [103]. Bedada et al. used piperine, resveratrol, and quercetin to test their effects on CYP2E1-mediated hydroxylation of the probe substrate chlorzoxazone in healthy volunteers. They showed that all three compounds alter the pharmacokinetics of chlorzoxazone through CYP2E1 inhibition [104]. Furthermore, the authors’ goal was to seek out more potential drugs to prevent hepatoxicity for severe alcohol drinkers. Leclercq et al. found that watercress, a cruciferous vegetable, inhibits chlorzoxazone metabolism, suggesting a potential solution for alcohol drinkers to prevent further liver damage [103,104,105]. However, four years later, the same group determined that watercress-induced inhibition of chlorzoxazone is minimal and, therefore, watercress has no significant effect on the elimination of ethanol by CYP2E1 inhibition [106]. This discrepancy is mainly because in the later study watercress was ingested 11 h prior to chlorzoxazone to study its preventive effect, while in the former studies they were given simultaneously. Thus, although watercress has the ability to inhibit CYP2E1, it does not alter overall chlorzoxazone distribution and urinal clearance. In addition, while several drugs inhibit CYP2E1 activity in vitro, some compounds and various disease states show opposite effects in humans and upregulate enzymatic activity. Recently, Sun et al. utilized aniline as a probe to compare hepatic CYP2E1 activity and found that prenatal ethanol exposure (4 g/kg/day) in male rat offspring inhibits CYP2E1 and 2D1 activities [107]. Additionally, a cocktail probe method was employed to examine the effects of Aidi (ADI) injection (a traditional Chinese medicine used for chemotherapy for various tumors) on various CYP activities in normal and liver cancer rats [108]. The authors observed that ADI can suppress activities of several CYP enzymes, including CYP2E1, and may be used in combination with other therapeutic regimens. Moreover, an active constituent of Garcinia indica and Garcinia cambogia, Garcinol, causes herb-drug interaction by inhibiting CYPs involved in drug metabolism [109]. In a previous study by Trousil et al., the authors included healthy controls and ovarian cancer patients, and gave them caffeine, chlorzoxazone, dextromethorphan, and omeprazole as in vivo probes to analyze CYP activity. CYP2E1 activity was upregulated and reduced in groups that received hydroxychlorzoxazone/chlorzoxazone and omeprazole, respectively [110]. Table 1 depicts a summary of clinical research probing CYP2E1-related interactions.

Wang et al. observed increased CYP2E1 activity in obese type-II diabetic patients with increased expression of CYP2E1 mRNA [124]. Loizou et al. and Oneta et al. both reported quick induction of CYP2E1 activity after acute alcohol consumption in healthy volunteers [112]. In another experimental group, Loizou et al. also noticed inhibition of CYP2E1 from DAS, a garlic extract, and proposed that regular consumption of this compound could reduce CYP2E1 activity [113,114]. Mitra and colleagues tested disulfiram to inhibit CYP2E1 activity to prevent the formation of hydroxylamine, a toxic metabolite of dapsone [113]. Interestingly, Frye et al. and Manyike et al. found similar results by using disulfiram on the metabolism of APAP [122]. Frye et al. also did similar studies and found similar DDI between disulfiram and vesnarinone [120,121].

## 5. Extracellular Vesicles (EVs) and CYP2E1

**Figure 1:**
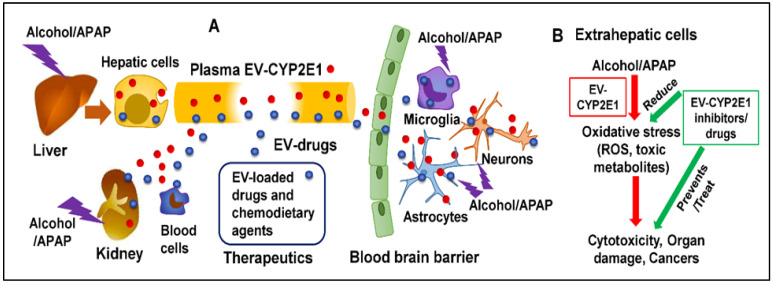
Schematic representation (**A**) and mechanisms (**B**) of CYP2E1 induction by alcohol/APAP. Encapsulated CYP2E1 (EVs-CYP2E1) is packaged in EVs from hepatic and extrahepatic cells, released into plasma, and circulated in periphery and CNS cells. Alcohol/APAP exposure to these cells causes oxidative stress followed by cytotoxicity, organ damage, and cancers. Attenuation of these outcomes is remedied by EVs-loaded drugs or nutraceutical agents circulating via plasma. Compounds loaded in EVs enter into hepatic/extrahepatic cells to reduce oxidative stress and protect or reverse alcohol/APAP-induced damage.

EVs are small extracellular nanovesicles (30–200 nm) that are produced by many cell types and are secreted in biofluids such as plasma, cerebrospinal fluids, urine, saliva, and milk [123]. EVs play a vital role in intracellular and intercellular communication via semi-selective packaging and transport of biomolecules such as miRNAs, mRNAs, proteins, and other biological cargo to neighboring cells or distant tissues via biofluids [30,31,32]. EVs have a unique ability to transport biomolecules and influence the pathophysiology of recipient cells. EVs in plasma are secreted from a variety of tissues, especially from the liver, upon xenobiotic exposure [125,126,127,128]. Thus, EVs are likely to play a critical role in modulating xenobiotic-induced toxicity in various extrahepatic tissue systems via EV-based transport of biomolecules [127]. In this section, we he role of EVs in cell-cell communications, especially intercellular communication. Figure 1 depicts the mechanism of CYP2E1 induction by alcohol/APAP packaging of CYP2E1 in EVs and the secretion into plasma. The EVs containing CYP2E1 subsequently circulate via plasma into distant cells, including brain cells, and cause cellular toxicity when exposed to alcohol and APAP.

### 5.1. Extracellular Vesicular CYP2E1

Kumar’s group reported the presence of various relevant physiologically and pharmacologically active CYP enzymes in human plasma EVs, including CYP2E1, as well as in monocytic cell-derived EVs [33,129,130,131]. In these studies, EVs were isolated first by filtering plasma through a 0.22-micron filter to remove large vesicles (>200 nm), followed by precipitation or ultracentrifugation methods. There are many methods for EV isolation, e.g., precipitation, ultracentrifugation, gel filtration, and their combination. The International Society of Extracellular Vesicles recommends a suitable method for EV isolation based on availability of the samples and need for studies, which have no or minimal interference with the outcomes. They observed that plasma-EVs from healthy subjects packaged metabolically active CYP2E1, and the level of CYP2E1 mRNA was 1000-fold higher when compared to other plasma CYP enzymes. Interestingly, the authors reported higher levels of CYP2E1 level in plasma EVs than the amounts seen in hepatic and extrahepatic (monocyte) EVs [33]. However, elevated CYP2E1 levels may not correspond with a similar increase in CYP2E1 activity. Thus, future studies are required to simultaneously investigate CYP2E1 levels and their activity for a prudent conclusion. Furthermore, Cho et al. demonstrated an increase in the total number of EVs and CYP2E1 expression in alcoholics and alcohol-fed animal models [34]. In this study, EVs were isolated using filtration through a 0.22-micron filter followed by an ultracentrifugation method. The role of CYP2E1 was demonstrated using CYP2E1-null mice and the CYP2E1 inhibitor chlormethiazole, which stopped the EV-induced toxic effects in the presence of alcohol. Moreover, CYP2E1 activity increased oxidative and endoplasmic reticulum stress, contributing to further vesicular packaging of CYP2E1. This EVs-CYP2E1 complex could potentially act as a biomarker for liver damage from long-term alcohol exposure. Plasma EVs CYP2E1 levels, at least in part, associate with alcohol-induced toxicity [35]. Future studies are needed to investigate the role of plasma EV CYP2E1 in alcohol-induced or drug-induced toxicity by blocking synthesis and packaging of CYP2E1 in EVs or selectively inhibiting plasma EV CYP2E1.

### 5.2. CYP2E1 in Cell-Cell Interactions

Intracellular signaling of EVs induced by the metabolic processing of alcohol leads to hepatitis, alcoholic-associated liver disease, and liver fibrosis [132]. Additionally, EV-based intercellular communication induced by alcohol could lead to damage to the immune, heart, pancreas, epithelial, and endothelial cells. Cho et al., have reported that CYP2E1-containing EVs, obtained from mice with APAP-induced liver injury, can cause hepatotoxicity in the recipient’s naïve primary hepatocytes [32]. Furthermore, treatment of the EVs obtained from these mice caused elevation of plasma ROS in mice and increased expression of proteins associated with apoptotic signaling pathways such as phospho-JNK/JNK, Bax, and cleaved caspase-3 in the recipient hepatocytes. Similarly, several studies have demonstrated the role of liver-derived EVs containing CYP2E1 upon alcohol exposure on hepatic and extrahepatic toxicities via intra-cellular and inter-cellular communications, especially between hepatocytes and immune cells [133,134].

Rahman et al. demonstrated that both human and murine plasma EVs cargos, specifically CYP2E1, play a role in exacerbating xenobiotic-induced toxicity in hepatic and extrahepatic cells [35,36,135]. EVs isolated from the plasma of healthy individuals and alcohol-exposed mice further increased alcohol-and APAP-induced toxicities in hepatic and monocytic cells. Alcohol-exposed mice also showed greater levels of plasma EVs CYP2E1 and decreased levels of oxidative defense and antioxidant enzymes. Toxicity in this study was reduced by the use of CYP2E1 siRNA and a CYP2E1 selective inhibitor, DAS [135]. These recent findings suggest the role of EVs CYP2E1 in intracellular and intercellular interactions, and their effects on xenobiotic-induced toxicity. In separate studies using human subjects, Kodidela et al. demonstrated the circulation of several cell-cell communication factors (various proteins, neuronal factors, inflammatory cytokines, and chemokines) in the plasma of alcohol drinkers [136,137]. In this study, EVs were isolated using filtration with a 0.22-micron filter followed by use of a double isolation commercially available precipitation kit.

## 6. Authors’ Perspectives

CYP2E1 is a druggable target to reduce the incidence of alcohol- and APAP-induced toxicity and drug-drug and alcohol-drug interactions [35]. However, there are currently no commercially available small molecule drugs that can specifically inhibit CYP2E1. Therefore, there is a need to design a novel treatment strategy using selective CYP2E1 inhibitors for alcohol and APAP overdoses. There are several reports on the use of DAS, an active ingredient of garlic extracts, as a selective CYP2E1 inhibitor [138,139]. In addition to CYP2E1 inhibition, DAS has many therapeutic properties including anticancer, antioxidant, antivirals, and anti-inflammatory activities [139]. Further, DAS has been used as a supplement to prevent and treat these diseases and conditions. Despite having many therapeutic properties, the use of DAS is restricted due to its inherent toxicity [139]. DAS is also a substrate of CYP2E1, which is metabolized into toxic metabolites DADS and diallyl trisulfide (DATS) [140]. These metabolites cause oxidative stress and cellular toxicity. Therefore, there is a critical need for replacing DAS with a safer CYP2E1 inhibitor. Rahman et al. designed several DAS analogs and conducted a thorough analysis of CYP2E1 inhibition kinetics [140,141]. Out of six selected DAS analogs, thiophene, allyl methyl sulfide, and diallyl ether appeared to be less toxic than DAS in in vitro assays. These compounds were also better than DAS in rescuing CYP2E1-mediated alcohol-induced and APAP-induced toxicities [142]. Research by Rahman et al. revealed CYP2E1 inhibitors as superior alternatives to DAS, which have the potential to be used in combination/preventive therapy for alcohol-induced and APAP-induced toxicities. Comprehensive pharmacokinetic and pharmacodynamic evaluations of these analogs in animal models are needed before realizing their potential in human applications.

The role of plasma EVs in cell-cell communication is becoming extremely important to understand the various physiological effects in disease pathologies and drug-induced organ toxicities, especially upon alcohol and APAP exposures [136,137,143]. CYP2E1 encapsulated in EVs is likely to be secreted from the liver and then transported to extrahepatic cells where metabolism of xenobiotics and endogenous drugs can occur. It is, therefore, possible that EV CYP2E1 performs a critical role in drug-drug and alcohol-drug interactions, not only in hepatic cells but also in distant extrahepatic cells. These EVs can also infiltrate into the CNS and deliver CYP2E1 to brain cells such as macrophages, microglia, astrocytes, and neurons (Figure 1). Delivery of CYP2E1 to these cells has the potential to increase the local concentration of CYP2E1, which would further increase the metabolism of alcohol and APAP causing alcohol-induced and APAP-induced brain toxicity. Concurrent use of alcohol and APAP is also likely to cause adverse interactions with other drugs that are taken simultaneously. Kumar et al. did not observe detectable levels of cytochrome P450 reductase (CPR) in plasma EVs [33]. However, brain cells have abundant CPR levels, which may be sufficient to metabolize alcohol [144,145]. Further, Rowland and colleagues have recently reported the presence of peptides and mRNA originating from various CYP and CPR in exosomes isolated from human plasma. Future studies are required to determine the quantity of these enzymes in plasma-derived EVs. Further studies are also needed to address whether increased plasma EV CYP2E1 levels result from the increased expression of hepatic or non-hepatic CYP2E1, synthesis of EVs, packaging of CYP2E1 in EVs, and/or secretion of CYP2E1-containing EVs in plasma. To address the role of EV CYP2E1 in distant cells/organs, especially in the brain, it is important to study whether EV CYP2E1 can efficiently circulate via plasma, cross the BBB, and be delivered to brain cells. Currently, it is also difficult to distinguish the effects of locally expressed CYP2E1 vs. cargo from EVs. There is also a lack tools such as an inhibitor for CYP2E1 packaging in EVs and an inhibitor to block EV secretion from the cells. Thus, to fully examine the role of EV CYP2E1 in cell-cell interactions, especially with respect to the brain, these limitations need to be overcome through future studies.

EVs, being similar to that liposomes and niosomes, are actively being investigated to develop novel drug delivery systems, especially across the BBB [135,142]. Zhuang et al. used EVs to encapsulate curcumin, a popular anti-inflammatory compound, to target brain tissue in mice, which efficiently crossed the BBB and selectively delivered curcumin to brain cells [129,130]. From the perspective of xenobiotic-induced liver damage, Nojima et al. used EVs as a drug delivery system to encapsulate sphingosine-1-phosphate, which was successfully delivered to hepatocytes and showed increased cell proliferation and tissue recovery [146]. Furthermore, Wu et al. found that EVs from hepatocytes were able to promote hepatocyte proliferation and liver recovery [147]. Since EVs have the capability of encapsulating small molecular chemicals, it is reasonable to hypothesize that EVs present in plasma, or derived from liver cells, could be used to package DAS analogs, allyl methyl sulfide, diallyl ether, and 2- prop-2-enoxyacetamide. EV-encapsulated drugs would not only reduce alcohol- and APAP-induced toxicity in hepatic and extrahepatic cells, but also suppress HIV in the CNS reservoirs. Nonetheless, before further investigating the role of EV-encapsulated CYP2E1 inhibitors, the challenges (loading, drug compatibility, toxicity profile) with EVs as a drug delivery system will need to be addressed.

Although there is a potential for EVs in drug delivery, EVs-based therapy will not reach patients without regulatory approval. Since there have been unsatisfactory outcomes of therapeutics for cancer, liver and cardiovascular diseases, EV-based therapy might be eligible for facilitated regulatory pathways (FRPs) [148]. Researchers are actively testing techniques to produce large quantities of EV-based drug products in compliance with current good manufacturing practices (cGMP) [149,150]. To mimic stem cell therapy, the delivery formulation needs to be sterile, injectable, and endotoxin-free [130]. Pachler et al. described their cGMP protocol for manufacturing EVs from human mesenchymal stromal cells in Austria, where such production is regulated by the European Medicines Agency (EMA) [151,152,153]. Harting et al. used a tangential flow filtration system to extract EVs from cells based on size difference [149,150]. This method is highly scalable and compliant with cGMP [154].

## Figures and Tables

**Table 1:** Summary of clinical research investigating CYP2E1 and related drug-drug and alcohol-drug interactions.

Reference	Condition/Drug 1	Effect	Drug 2: Induced Toxicity
[111]	Diabetes	Induction	Chlorzoxazone
[112]	Ethanol	Induction	Chlorzoxazone
[113]	Ethanol	Induction	Chlorzoxazone
[114]	Ethanol	Induction	N/A
[115]	Isoniazid	Induction	Chlorzoxazone
[116]	Chlormethiazole	Inhibition	Chlorzoxazone
[117]	Chlorzoxazone	Inhibition	Midazolam
[118]	Chlorzoxazone	Inhibition	Midazolam
[119]	Diallyl Sulphide	Inhibition	Chlorzoxazone
[113]	Disulfiram	Inhibition	Acetaminophen
[120]	Disulfiram	Inhibition	Acetaminophen
[121]	Disulfiram	Inhibition	Dapson
[122]	Disulfiram	Inhibition	Vesnarinone
[123]	Piperine	Inhibition	Chlorzoxazone
[104]	Quercetin	Inhibition	Chlorzoxazone
[105]	Resveratrol	Inhibition	Chlorzoxazone
[103]	Watercress	Inhibition	Chlorzoxazone
[106]	Watercress	Inhibition	Ethanol

## Data Availability

Not applicable.
